# VDR polymorphism, gene expression and vitamin D levels in leprosy patients from North Indian population

**DOI:** 10.1371/journal.pntd.0006823

**Published:** 2018-11-27

**Authors:** Itu Singh, Mallika Lavania, Vinay Kumar Pathak, Madhvi Ahuja, Ravindra P. Turankar, Vikram Singh, Utpal Sengupta

**Affiliations:** Stanley Browne Laboratory, The Leprosy Mission Trust India, The Leprosy Mission Community Hospital, Nand Nagari, New Delhi, Delhi, India; Hospital Infantil de Mexico Federico Gomez, UNITED STATES

## Abstract

**Background:**

Leprosy is a chronic infectious disease caused by *Mycobacterium leprae* and mainly affects skin, peripheral nerves. Vitamin D receptor (VDR) gene polymorphism has been found to be associated with leprosy. Vitamin D has been shown to control several host immunomodulating properties through VDR gene. Vitamin D deficiency was also found to be linked to an increased risk for several infections and metabolic diseases.

**Objective:**

In the present study, we investigated the association of VDR gene polymorphism, mRNA gene expression of VDR and the vitamin D levels with leprosy and its reactional states.

**Methodology:**

A total of 305 leprosy patients consisting of tuberculoid (TT), borderline tuberculoid (BT), borderline lepromatous (BL), lepromatous leprosy (LL), as well as 200 healthy controls were enrolled in the study. We identified single nucleotide polymorphisms (SNPs) of VDR Taq1, Fok1 and Apa1, as well as the expression of VDR mRNA gene using PCR-based restriction fragment length polymorphism (RFLP) analysis and real-time PCR respectively. We also performed ELISA to measure vitamin D levels.

**Result:**

We observed that SNP of VDR gene (Fok1 and Taq1) are associated with the leprosy disease. The allelic frequency distribution of T and t allele (p = 0.0037), F and f allele (p = 0.0024) was significantly higher in leprosy patients and healthy controls. ff genotype of Fok1 was found to be associated with leprosy patients [p = 0.0004; OR (95% CI) 3.148 (1.662–5.965)]. The recessive model of Fok1 genotype was also found to be significantly associated in leprosy patients in comparison to healthy controls [p = 0.00004; OR (95% CI) 2.85 (1.56–5.22)]. Leprosy patients are significantly associated with t-F-a haplotype. Further, VDR gene expression was found to be lower in non-reaction group compared to that of reaction group of leprosy and healthy controls. Paradoxically, we noted no difference in the levels of vitamin D between leprosy patients and healthy controls.

**Conclusion:**

Blood levels of vitamin D do not play any role in clinical manifestations of any forms of leprosy. ff genotype of Fok1 and tt genotype of Taq1 was found to be associated with leprosy per se. Association of t-F-a haplotype with leprosy was found to be significant and could be used as a genetic marker to identify individuals at high risk for developing leprosy. VDR gene expression was lower in TT/BT and BL/LL groups of leprosy in comparison to that of healthy controls.

## Author summary

Present study was carried out to find out the association of vitamin D receptor (VDR) gene polymorphism, mRNA gene expression of VDR gene and level of vitamin D with leprosy reactions and leprosy patients. Surprisingly, level of vitamin D in leprosy patients was not found to be associated with the disease and its manifestations. VDR genotypes (Fok1 and Taq1) were found to be associated with leprosy patients. t-F-a haplotype was significantly associated with leprosy patients. Gene expression of vitamin D receptor was lower in leprosy patients in comparison to healthy controls.

## Introduction

Leprosy is a chronic, debilitating infectious disease caused by *Mycobacterium leprae* that involves skin, peripheral nerves and mucous membranes. At one pole is tuberculoid (TT) leprosy, characterized by strong Th1 immunity with few bacilli in skin lesions and at the opposite pole is lepromatous (LL) leprosy, characterized by large number of bacilli scattered throughout the skin lesions expressing high Th2 type of immunity [1]. In between these two poles the disease is manifested in a spectrum as borderline tuberculoid (BT), borderline (BB) and borderline lepromatous (BL) determined by the immune status of the host. Two types of reactions are commonly observed in leprosy patients. Type 1 or reversal reaction (T1R) mainly occurs after the initiation of treatment in patients at the borderline pole of the leprosy spectrum (BL, BB, and BT) with up-regulation of the cell mediated immunity (CMI) and consequently shifting from a Th2 type to a Th1 type of immune response. Type 2 reaction (T2R) or erythema nodosum leprosum (ENL) occurs in BL/LL group of leprosy patients and is more commonly seen in patients with high bacterial load along with high level of *M*. *leprae* antibody resulting in deposition of immune complexes and complement [2].

Host immune system plays a vital role in monitoring and controlling the spread of infection. Genetic polymorphisms in the immune reactive genes have been involved in making a person more susceptible or resistant to infections. Genetic studies have indicated that association of HLA [3] and some non-HLA genes are responsible for susceptibility to leprosy phenotypes that occurs upon infection [4, 5]. Vitamin D Receptor (VDR) is a member of the nuclear receptor family and it controls transcriptional responses and regulate micro-RNA directed post-transcriptional mechanisms for initiation of an effective immune response. Hence, vitamin D facilitates several immuno-modulatory properties through VDR. A single dose of vitamin D supplementation has been reported to enhance the immunity to mycobacteria [6]. VDR is present on many immune cells like macrophages, dendritic cells, T and B lymphocytes [7] and after stimulation it plays a dynamic role in host immune response [8]. It has been reported earlier that among the allelic genotypes of Taq1 polymorphisms genotypes tt homozygous tend to produce Th1-type responses while TT homozygous produce a Th-2 type response [9]. Frequency and distribution of VDR gene (Taq1, Fok1 and Apa1) in healthy individuals of Indian population was reported earlier [10]. Previously in a Bengali population Taq1 polymorphism was found to be associated with leprosy [9]. It has been shown that The VDR Fok1 ff genotype and Apa1 AA, Aa genotype and haplotype T-f-a, T-F-A were positively associated with leprosy in South-Indian population [11].

Vitamin D deficiency was found to be linked with increased risk of some infectious and metabolic diseases [12, 13]. It has been documented that hypercalcemia suppressed 1, 25-dihyroxyvitamin-D3 (1,25OHD) levels [14]. It was also reported that serum 1,25OHD and calcium levels remain elevated in patients with leprosy [15]. However, no differences in serum 1,25OHD levels between leprosy patients and normal individuals have also been reported [16]. Further, vitamin D in massive doses was used along with sulfones in the treatment tuberculoid leprosy which modified the clinical course of the disease [6]. Recently, Mandal et al (2015) reported significantly lower levels of vitamin D in leprosy patients compared to their household controls [17]. In the light of above-mentioned facts, we hypothesize that there might be an association between vitamin D level with the leprosy and that VDR gene polymorphism may show association with leprosy and also VDR mRNA gene expression may be associated with the disease status. Since, osteoporosis is a major problem in lepromatous forms of leprosy along with conflicting reports on vitamin D levels in leprosy, the present study has been undertaken to find out the associations of vitamin D level and mRNA VDR gene expression VDR gene in leprosy patients and in reaction (T1R and T2R) group of leprosy. Further, an attempt has also been made to investigate the association of VDR gene polymorphism, if any, with leprosy patients with and without reactions.

## Materials and methods

### Ethics statement

This study was approved by The Leprosy Mission Trust India (TLMTI) human ethics committee following the guidelines of the Indian Council of Medical Research (ICMR) in a meeting held on 29^th^ August, 2014 with REC No. EC 5/14/a, Protocol No. 1/5/14. All the adult participants (age >18 years) who agreed to participate in the study with written informed consents were enrolled. In the case of minors (age 10–18 years) written informed consent was obtained from their parents/guardians on behalf of all child participants.

### Participants

Leprosy patients were recruited from the outpatient department (OPD) of TLM Community Hospital, Shahdara, Delhi. A total of 305 leprosy patients [NR (218) (TT/BT (n = 128), BL/LL (n = 90)), T1R (n = 74) and T2R (n = 13)] were enrolled in the study. Patients were diagnosed clinically by experienced dermatologists and classified on Ridley-Jopling scale (1966) [18]. We recruited leprosy patients of TT/BT and BL/LL group without active reaction and no history of leprosy reactions in the past years. T1R group is characterized by development of acute inflammation in skin lesions/nerves/both. T2R is a serious systemic illness with inflamed nodules, papules. Neuritis occurs in both types of leprosy reactions and it is major cause of disability in leprosy patients. Leprosy cases with HIV sero-positive, tuberculosis (TB), diabetes and any other dermatological and immunosuppressive disorders were excluded from the study. Healthy individuals (n = 200) of the same locality with similar social and financial backgrounds were enrolled as healthy controls (HCs). HCs having family history of leprosy or TB and HIV were also excluded from the study. The demographic characteristics of the leprosy patients and healthy controls were tabulated in Table 1.

**Table 1:** Demographic characteristics of leprosy patients and healthy controls.

Characteristics		Leprosy Patients (n = 305)	Healthy Controls (n = 200)	P value
**Age (Mean ± SD)**		35.01 ± 13.95	32.69 ± 10.95	0.096^a^
**Gender (%)**	Male	194 (63.60)	126 (63)	0.89^b^
	Female	111 (36.39)	74 (37)	
**TT/BT (%)**		202 (66.23)	-	
**BL/LL (%)**		103 (33.78)	-	
**T1R (%)**		74 (24.26)	-	
**T2R (%)**		13 (4.26)	-	

^a^ Mean age of leprosy patients and healthy controls was compared by Mann-Whitney U test

^b^ Gender of leprosy patients and healthy controls was compared by Mann-Whitney U test

### Sample collection and analysis

Peripheral blood samples were collected from all the participants and sera were separated and stored at -20°C until further processing.

### DNA extraction

DNA was extracted from whole blood using DNeasy blood and tissue kit (Qiagen, Hilden, Germany) according to manufacturer’s instructions. DNA concentration was estimated on a UV Spectrophotometer (Shimadzu Inc. Japan).

### RNA extraction

RNA was extracted from whole blood using RNeasy blood kit (Qiagen, Hilden, Germany) according to the manufacturer’s instructions. RNA concentration was estimated on a UV Spectrophotometer (Shimadzu Inc. Japan).

### Genotyping of VDR

A total of 505 samples were included for genotyping. Genotyping of VDR Taq1, Fok1 and Apa1 polymorphisms was done by PCR-RFLP method using specific primers [19, 20, 21] and restriction enzymes.

### Taq1 polymorphism

The PCR was done with some minor modifications using published protocol by Bid et al (2005) [10]. Briefly, PCR cycling conditions were consisted of 35 cycles of 94°C for 60s, 63°C for 60s and 72°C for 2 min each, preceded by a single cycle of 94°C for 5 min and followed by a single cycle of 72°C for 10 min. The PCR product (740 bp) was digested with Taq1 (NEB) restriction enzyme at 65°C for 20 min inactivated at 80°C for 20 min. Digested products were either of the homozygous TT (absence of specific restriction site) of 245 bp and 495 bp or homozygous tt yielding 205, 245, 290 bp and heterozygous Tt yielding 205, 245, 290, 495 bp [10].

### Fok1 polymorphism

PCR cycling conditions were of 35 cycles at 94°C for 30s, 61°C for 30s and 72°C for 1 min each, preceded by a single cycle of 94°C for 5 min and followed by a single cycle of 72°C for 10 min. PCR product (265 bp) was digested with Fok1 (NEB) restriction enzyme, reaction was incubated at 37°C for 1 hr and inactivated at 65°C for 20 min. Digested products were of FF (265 bp), ff (196 and 69 bp) and Ff (265, 196 and 69 bp).

### Apa1 polymorphism

PCR cycle conditions were initiated with denaturation at 94°C for 4 min followed by 35 cycles at 94°C for 30s, 65°C for 30s and 72°C for 2 min and with final extension at 72°C for 10 min. Amplified product (2000 bp) was digested with restriction enzyme Apa1 (NEB) for 25°C for 20 min and was inactivated at 65°C for 20 min. Digested product was of AA (2000 bp), aa (1700, 300 bp) and Aa (2000, 1700 and 300 bp).

### Vitamin D level

A total of 119 participants consisting of HC (n = 31), TT/BT (n = 26), BL/LL (n = 21), T1R (n = 28) and T2R (n = 13) were included in the study. Estimation of serum vitamin D levels was outsourced from M/s Thyrocare, India. It has been done by fully automated chemiluminescence immunoassay by using ADVIA Centaur.

### Gene expression of vitamin D mRNA

cDNA was constructed from 1 μg of total RNA from each of the sample using cDNA Synthesis Kit (NEB #6300S, New England Biolabs Inc) according to the manufacturer’s instructions. Briefly, 1μg of total RNA was mixed with Random Primer mix and nuclease free water. RNA was then denatured at 70°C for 5 min and followed by the addition of 1×M-MuLV Reaction Mix containing buffer, Magnesium ions, dNTPs and 1×M-MuLV Enzyme Mix containing 0.5 units/μl of Reverse Transcriptase and RNase Inhibitor. Temperature cycle conditions were 25°C for 5 min, 45°C for 1 hr and inactivation of enzyme was carried out at 80°C for 5 min.

cDNA corresponding to VDR transcript was amplified on Rotor Gene-Q (Qiagen Inc. USA) real-time PCR machine using primers and reaction conditions as published earlier [22]. Briefly, 10μl of Rotor-Gene SYBR Green PCR Master Mix (Qiagen, Cat No. 204074), 200 nM concentration of each of the forward and reverse primers for VDR and 200 ng of cDNA (total of 25 μl reaction mix) are amplified in Rotor-Gene Q with cycling conditions as 95°C for 10 min (initial denaturation and activation of enzyme) followed by 40 cycles of 95°C for 10 s, annealing at 61°C for 15s and elongation at 72°C for 20 s. The fluorescence was acquired on green channel during the annealing step. A melting curve analysis was performed by heating the amplicons from 65°C to 95°C with 1°C/s raise in temperature at each step. The mRNA expression levels were normalized by using GAPDH (glyceraldehyde 3 phosphate dehydrogenase) mRNA as a house-keeping gene. The threshold fluorescence values were normalized to those of GAPDH values. The mRNA expression levels were calculated after determining the primer efficacy for all the genes using Pfaffl Method [23] by a standard curve with a 6-fold dilution of cDNA from 1 μg/reaction to 10 ng/reaction. Melting curve analysis was performed to determine the integrity of the amplification and to rule out primer–dimer formation. The percentage efficiency of the primers from the standard graphs were determined to be in the order of 98% for GAPDH, 93% for VDR. The fold difference in expression was calculated based on the below formula:

$$\text{Ratio}={\left({\text{E}}_{\text{target}}\right)}^{\Delta \text{C}{\text{T}}_{\text{target}}(\text{Control}–\text{Sample})}/{\left({\text{E}}_{\text{ref}}\right)}^{\Delta \text{C}{\text{T}}_{\text{ref}}(\text{Control}–\text{Sample})}$$

Individual expression ratios using the above formula were computed for all the subjects taking the average ratio of HC as the control value for all the cases.

### Statistical analysis

The statistical analysis was performed using Graph-Pad Prism software (Version 6). Chi-square test was done for investigating Hardy-Weinberg equilibrium and for comparing the genotypes and alleles frequencies between the controls and different phenotypes of leprosy. Odds ratio (OR) and 95% confidence interval (CI) were computed for the association between genotype and leprosy. An individual genotype analysis was performed using logistic regression model corrected by sex for the significant detected genotype by SNPstat software. Haplotype analysis and association was carried out using online software SNPstat (https://www.snpstats.net/snpstats/preproc.php) [24]. For genetic association study we adjusted the threshold value (0.02) below which p-value is considered significant. The level of vitamin D and VDR gene expression among all the studied groups were analyzed by One-way ANOVA non-parametric Kruskal-Wallis test. Dunn’s multiple comparison test was used to find out the difference between healthy controls and groups of leprosy patients. For all analysis p value, less than 0.05 was considered as statistically significant. The real-time data was analyzed on Rotor-Gene Q Series Software (Software Version 2.0.2).

## Results

### Participants characteristics

A total of 305 leprosy patients [NR (218), T1R (n = 74) and T2R (n = 13)] were enrolled in the study. Healthy individuals (n = 200) from the same geographical area and having similar societal and financial background were included as healthy controls (HCs).

### Genotypic analysis of VDR gene polymorphisms

The allele and genotype distribution of each SNP (Taq1, Fok1, Apa1) of VDR gene was in agreement with Hardy-Weinberg equilibrium (p>0.05) in leprosy patients and healthy controls.

The frequency distribution of alleles, genotypes and association analysis is described in Table 2. A significant difference in the distribution of Taq1 position TT, Tt, tt (p = 0.0037) and Fok1 position FF, Ff and ff was observed (p = 0.0048) between leprosy patients and healthy controls. The allelic frequency distribution of T and t allele (p = 0.0037), F and f allele (p = 0.0024) was significantly different between leprosy and healthy controls. However, considering the difference in the frequency of tt genotype in leprosy patients (14.81%) and healthy controls (7.51%), genotypes were analyzed for significant association. The recessive model of Taq1 was found to be associated with leprosy patients [OR (95%CI) 0.47 (0.24–0.89, p = 0.016) (Table 2). ff genotype of Fok1 was found to be associated with leprosy patients [p = 0.0004; OR (95% CI) 3.148 (1.662–5.965)] (Table 2). The recessive model of Fok1 genotype was found to be significantly associated in leprosy patients in comparison to healthy controls [p = 0.00004; OR (95% CI) 2.85 (1.56–5.22)] (Table 2). No significant difference was observed in allele and genotype frequencies for Apa1 polymorphism between leprosy patients and healthy controls (p>0.02) (Table 2).

**Table 2:** Genotype and allele frequencies of VDR gene (Taq1, Fok1 and Apa1) polymorphism among leprosy patients and healthy controls.

Genotype/allele	Leprosy patients (n = 305)	Healthy controls (n = 200)	OR (95% CI)	P value of χ^2^ test
Taq1 (rs731236)	N = 297 (%)	N = 173 (%)		
HWE (p value)	1.0	0.59		
TT	111 (37.37)	85 (49.13)	Reference	0.0037*
Tt	142 (47.81)	75 (43.35)	0.6897 (0.4634–1.027)	0.069
tt	44 (14.81)	13 (7.51)	0.3858 (0.1954–0.7618)	0.0053
T allele	364	245		
t allele	230	101		0.0037
TT vs Tt+tt			0.6178 (0.4226–0.9032)	0.015
tt vs Tt+TT			0.47 (0.24–0.89)	0.016
Fok1 (rs2228570)	(N = 288)	(N = 185)		
HWE (p value)	0.12	0.44		
FF	137 (47.57)	71 (38.38)	Reference	0.0048*
Ff	132 (45.83)	83 (44.86)	1.213 (0.8157–1.805)	0.363
ff	19 (7)	31 (16.76)	3.148 (1.662–5.965)	0.0004
F allele	406	225		
f allele	170	145		0.0024
FF vs Ff+ff			1.457 (1.000–2.122)	0.049
ff vs Ff+FF			2.85 (1.56–5.22)	0.00004
Apa1 (rs7975232)	(N = 297)	(N = 160)		
HWE (p value)	0.8	0.09		
AA	124 (41.75)	56 (35)		0.2716*
Aa	138 (46.46)	86 (53.75)		
aa	35 (11.78)	18 (11.25)		
A allele	386	198		
a allele	208	122		0.3864
AA vs Aa+aa			1.331 (0.8936–1.983)	0.1619
aa vs Aa+AA			1.054 (0.5759–1.928)	1.00

*p value for 3X2 χ^2^ test for comparison among all the genotype frequencies for leprosy patients and healthy control.

No significant difference was observed for Taq1, Fok1 and Apa1 genotype in reactional groups of leprosy when compared with non-reactional group of leprosy (Table 3).

**Table 3:** Genotype frequencies of VDR gene (Taq1, Fok1 and Apa1) polymorphism among Type 1 reaction (T1R), Type 2 reaction patients (T2R) and Non-reaction (NR) leprosy patients.

Genotype/allele	Type 1 Reaction (T1R)	Type 2 Reaction (T2R)	Non-Reaction (NR)	OR (95% CI)	P value of χ^2^ test
Taq1	N = 70	N = 11	N = 216		
TT	25	5	81		0.64^a^, 0.75^b^
Tt	39	4	99		0.46^a^, 0.73^b^
tt	6	2	36		0.27^a^, 1.00^b^
TT vs Tt+tt				0.9259 (0.5282–1.623)^a^, 1.389 (0.4106–4.698)^b^	0.89^a^, 0.75^b^
tt vs Tt+TT				0.4688 (0.1886–1.165)^a^, 1.111 (0.2303–5.361)^b^	0.12^a^, 1.00^b^
Fok1	N = 69	N = 13	N = 206		
FF	28	8	101		0.24^a^, 0.71^b^
Ff	36	3	93		0.72^a^, 0.22^b^
ff	5	2	12		0.54^a^, 0.32^b^
FF vs Ff+ff				0.71 (0.4084–1.234)^a^, 1.663 (0.5264–5.256)^b^	0.27^a^, 0.41^b^
ff vs Ff+FF				1.263 (0.4284–3.723)^a^, 2.939 (0.5841–14.79)^b^	0.77^a^, 0.19^b^
Apa1	N = 72	N = 13	N = 212		
AA	28	3	93		0.94^a^, 0.33^b^
Aa	40	9	89		0.20^a^, 0.13^b^
aa	4	1	30		0.23^a^, 1.00^b^
AA vs Aa+aa				0.8143 (0.4716–1.406)^a^, 0.3839 (0.1027–1.435)^b^	0.49^a^, 0.16^b^
aa vs Aa+AA				0.3569 (0.1212–1.051)^a^, 0.1704 (0.02180–1.332)^b^	0.059^a^, 0.068^b^

P value for 3x2 χ^2^ test of comparison of overall genotype frequencies between ^a^T1R and NR,

^b^T2R and NR group of leprosy

### Haplotype association between the study groups

The frequency of haplotype t-F-a was significantly higher in leprosy patients in comparison to HC (p<0.0001) suggesting a positive association of this haplotype (Table 4) with the disease. No linkage disequilibrium was observed between SNPs tested.

**Table 4:** Haplotype frequency distribution among leprosy patients and healthy controls.

Haplotype	Leprosy Patients	Healthy Controls	P value
T-F-A	0.261	0.238	
t-F-A	0.214	0.149	0.41
T-F-a	0.162	0.219	0.18
T-f-a	0.111	0.131	0.21
t-f-A	0.094	0.11	0.46
T-f-A	0.078	0.118	0.088
t-F-a	0.066	0	<0.0001
t-f-a	0.014	0.035	0.27

### Level of vitamin D in the participants

**Fig 1:**
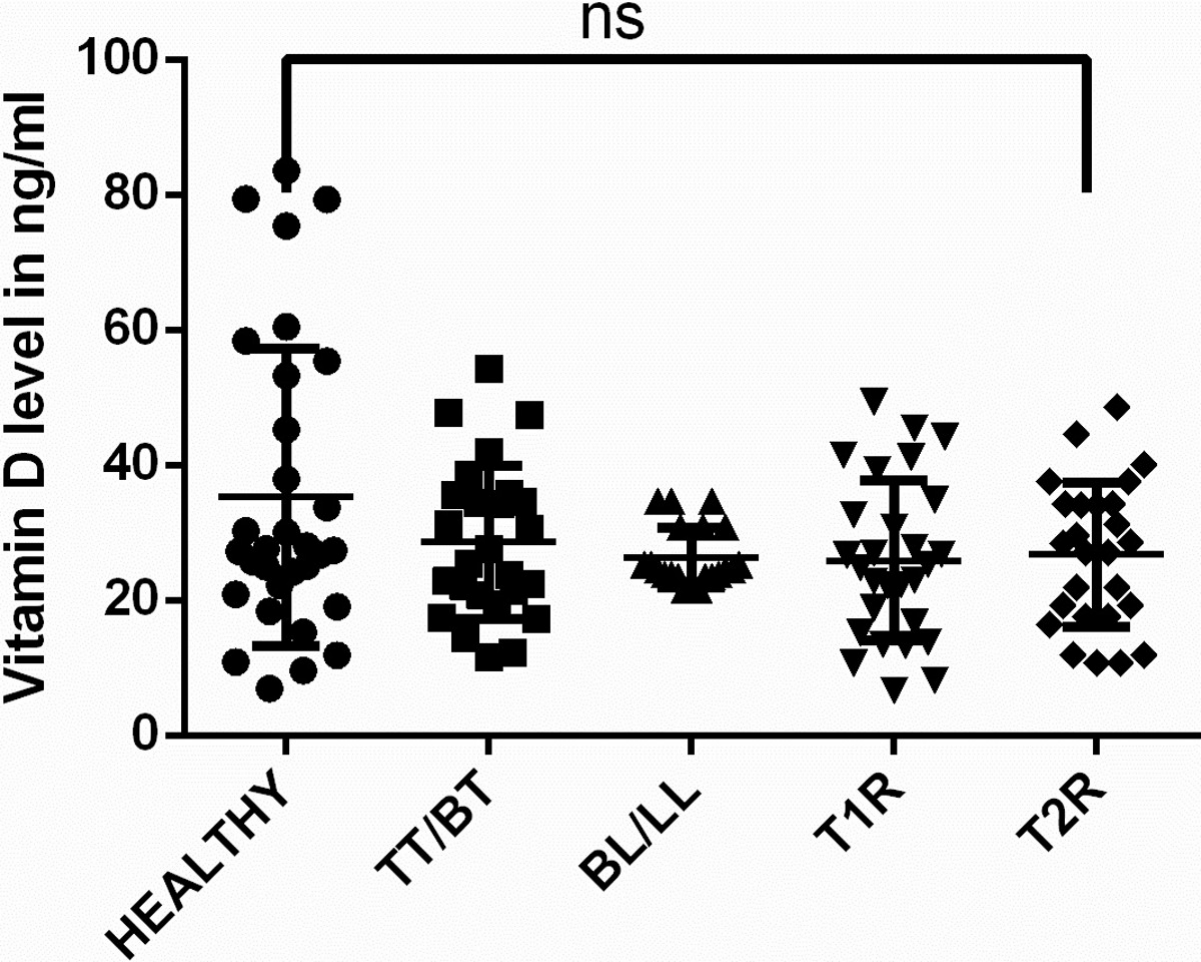
Graph showing levels of vitamin D in healthy controls and leprosy patients. mRNA expression profile of VDR gene in the study subjects.

Average levels of vitamin D among healthy controls and leprosy patients are shown in Table 5 and Fig 1. It is noted that there was no difference in the values of vitamin D levels amongst any of the groups (Fig 1). We observed average level of vitamin D in healthy controls (mean±SD = 35.30±22.02) and leprosy patients (mean ± SD = 26.91±10.89) is also not significantly different.

**Table 5:** Average level of vitamin D among healthy controls and leprosy patients.

	Healthy Controls (n = 31)	Leprosy Patients			
		TT/BT (n = 26)	BL/LL (n = 21)	T1R (n = 28)	T2R (n = 13)
**Mean level of Vitamin D in ng/ml ± Standard Deviation** **p value**	35.30±22.02	28.60±11.31 p = 0.54	26.37±4.436 p = 0.28	25.89±11.78 p = 0.16	26.78±10.67 p = 0.42

**Fig 2:**
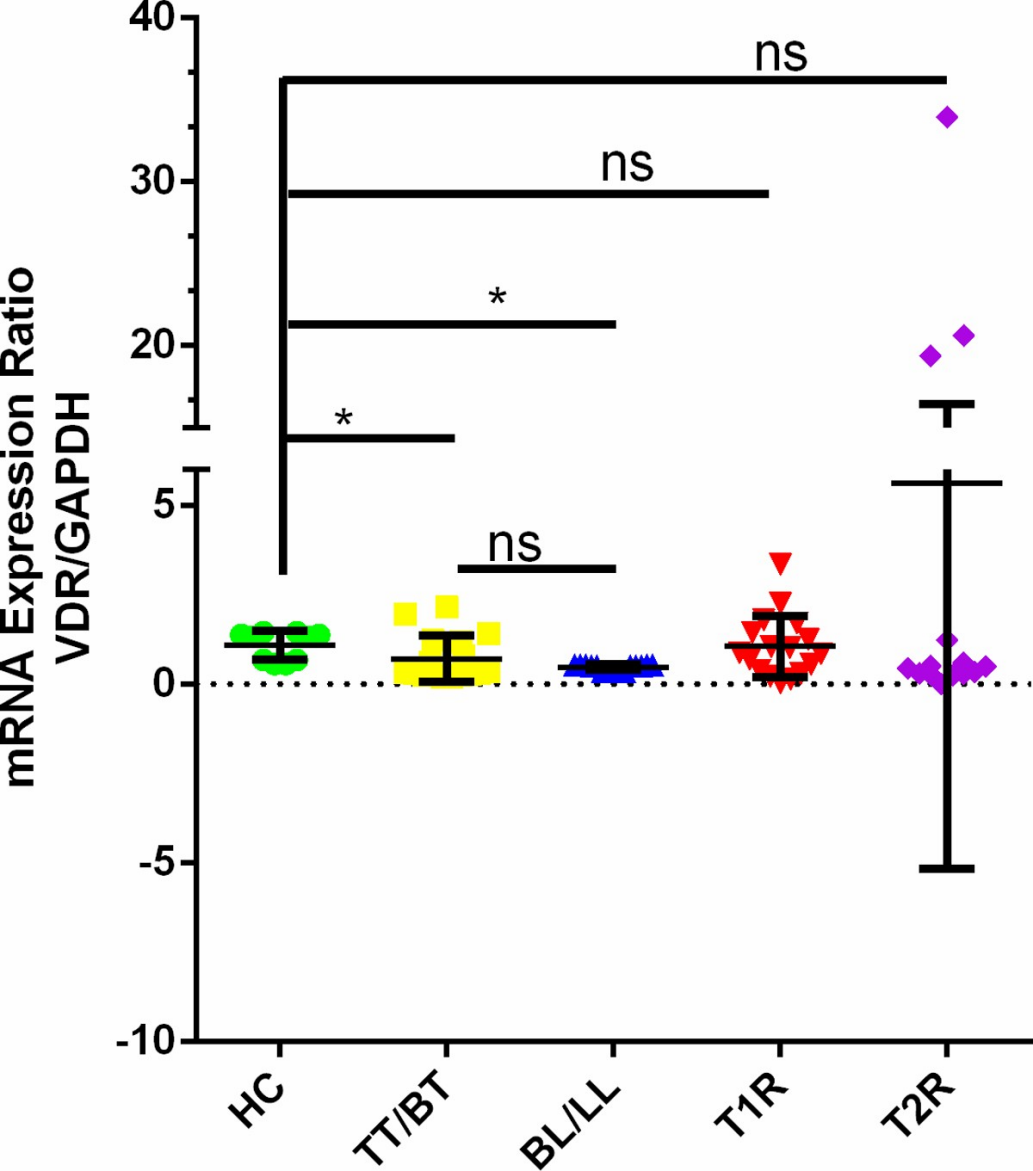
Comparative analysis of mRNA expression ratios of VDR/GAPDH in blood samples among the HC (n = 10), TT/BT (n = 17), BL/LL (n = 15), T1R (n = 17) and T2R (n = 13) using real-time PCR.

The mean mRNA expression ratios of VDR/GAPDH are identified to be significantly lower in TT/BT and BL/LL group of leprosy in comparison to the HC (TT/BT vs. HC, 0.702 vs. 1.076 and BL/LL vs HC, 0.462 vs 1.076 p < 0.05) (Fig 2). There was no significant difference observed between TT/BT and BL/LL groups of leprosy (TT/BT vs BL/LL, 0.702 vs 0.462 p>0.05). Highest mean mRNA expression ratio of VDR/GAPDH was observed in T2R group of leprosy however it is not significant in comparison to HC (T2R vs HC 5.62 vs 1.076, p>0.05) followed by T1R (1.044) (Fig 2). Further, when we compared mean mRNA expression ratio of VDR/GAPDH between non-reaction (NR) and T1R/T2R groups of leprosy, it was noted that the ratio is not significantly different between the groups (NR vs T1R 0.5894 vs 1.044, p = 0.05; NR vs T2R 0.5894 vs 5.62, p = 0.9529).

## Discussion

The association of SNPs of VDR gene (Taq1, Fok1 and Apa1), serum level of vitamin D along with VDR mRNA expression with the susceptibility to different phenotypes of leprosy was assessed in this study. It has been reported earlier that immunomodulatory role of vitamin D occurs through its receptor, VDR and it may help to promote appropriate innate immune responses [8]. Vitamin D has been shown to promote a T-cell shift from Th1 to Th2 [25] and transcription of antimicrobial peptides [26]. The polymorphisms in VDR gene (Taq1, Fok1 and Apa1) may affect mRNA stability which in turn affect protein levels and eventually may alter the balance of the production of the Th1 and Th2 related cytokines [25] which is a decisive factor for determining the clinical outcome of leprosy.

Present study has indicated some interesting findings. Firstly, in our study population mainly from North India Taq1 and Fok1 association with leprosy patients was observed and noted that tt and ff are significantly more prevalent in leprosy patients in comparison to HC. Though power of association being a limitation of the study our observations suggest that ff and tt genotypes might be associated with the susceptibility with leprosy. Similarly, in an earlier study, ff genotype was found to be associated with leprosy in South-Indian population [11]. In another study, authors observed that Ff genotype frequency was highest in North Indian healthy population [10]. While, Neela et al (2015) reported that allele A and genotype AA and Aa are associated with leprosy [11]. We have not found any significant association of Apa1 genotype of VDR gene with leprosy. No association of Apa1 polymorphism has been shown to be associated with leprosy in Nepalese population [27]. However, allele A of Apa1 was found to be positively associated with tuberculosis in African population [28]. However, Taq1 polymorphism was not found to be associated with leprosy in south Indian population [11]. Similarly, Taq1 polymorphism was found to be associated with leprosy in Mexican population and Bengali Indian population [29, 9]. It has also been reported that Taq1 polymorphism is associated with tuberculosis in Gambian population [30]. We also observed significant association of Taq1 polymorphism with leprosy. This difference of the associations might be due to different ethnic groups studied by the researchers. Further, we observed that t-F-a haplotype is associated with leprosy per se.

As we know that persons affected with leprosy have bone deformities and manifest osteoporosis hence, we estimated the plasma level of vitamin D in leprosy patients and healthy controls. The present study showed no significant difference in the levels of vitamin D across the spectrum of leprosy including reactional cases in comparison to HC. Similarly, Ribeiro et al (2007) noted same serum level of vitamin D in leprosy patients in comparison to healthy controls [16]. To the best of our knowledge there is no report on vitamin D level in leprosy patients in India except one reported by Mandal et al (2015) wherein leprosy cases with reactions were shown to have a lower levels of vitamin D compared to HC [17]. Differences which have been noted in the level of vitamin D in these studies might be due to differences in cultural behaviors, exposure to sunshine and dietary intake of vitamin D in the participants. It has been reported earlier that incubation of human macrophages and monocytic cell lines with vitamin D inhibit intracellular growth of *M*. *tuberculosis* [31]. The mechanism could be that treatment with vitamin D might increase the differentiation and cytotoxicity in macrophages. In the present set up, such in vitro studies are not possible to perform with *M*. *leprae*. There are many contentious reports showing association between vitamin D levels and tuberculosis [32, 33]. One recent study has reported the association of higher levels of vitamin D with active tuberculosis [34]. We suggest that sun exposure is the main source of vitamin D and most of the leprosy patients studied here having agricultural background were usually exposed to sun. Further, bone deformities and osteporotic changes in early and advanced stages of leprosy has already provided evidence that the bone damages are mainly due to direct invasion of bone by *M*. *leprae* [35, 36, 37, 38].

We further observed a significantly lower VDR/GAPDH mRNA expression ratio in TT/BT and BL/LL groups of leprosy in comparison to HC group. Highest VDR/GAPDH gene expression ratio was observed in T2R cases in comparison to all the phenotypes of leprosy. In contrast to the present study earlier it has been reported that VDR mRNA expression was significantly lower in T2R group of leprosy patients in comparison to HC group [17]. It has also been reported that mRNA expression for VDR in skin lesions of lepromatous patients is lower in comparison to that with BT/TT leprosy and in T1R [39]. We found significantly lower VDR mRNA expression in tuberculoid and lepromatous group of leprosy while we noted no significant difference in the level of vitamin D. Low level of gene expression VDR gene in leprosy patients in comparison to HCs might be associated with the VDR gene polymorphism found in leprosy patients and one study Selvaraj et al (2009) have reported that vitamin D has no effect on the mRNA expression of VDR in tuberculosis patients [40]. It has been shown earlier that ethnicity influenced the VDR expression and serum level of vitamin D but did not influence VDR expression and its function in South African healthy population [41]. However, it has been shown by Zella et al (2010) that vitamin D induced the accumulation of VDR in osteosarcoma cells [42]. All these findings suggest that vitamin D status is dependent on multiple factors and VDR mRNA expression is not only dependent on vitamin D status in an individual but also on expression of multiple factors. This study demonstrated for the first time in north Indian population that Taq1 and Fok1 genotypes are associated with the leprosy and t-F-a haplotype could be useful to detect high risk group for leprosy. Further, low VDR gene expression level in patients might be responsible for impaired immune response in leprosy. Further, genotype-based study with a larger number of samples would be useful in understanding their role in genetic risk factor in leprosy.

Present study demonstrated that SNP of VDR gene (Fok1 and Taq1) are associated with the leprosy per se and it might be responsible for lower VDR gene expression in leprosy. No association of Taq1, Fok1 and Apa1 with reactional group of leprosy was noted. t-F-a haplotype is significantly associated with leprosy per se. Haplotype analysis could be useful to screen the higher risk group of HCs and endemic population of leprosy patients. Surprisingly, no significant difference in the levels of vitamin D was noted amongst leprosy patients in comparison to healthy controls. VDR mRNA expression was found to be significantly lower in tuberculoid and lepromatous groups of leprosy. However, VDR mRNA expression was highest in T2R group amongst all the studied groups.
